# A184 EARLY SYMPTOM CONTROL WITH MIRIKIZUMAB IN PATIENTS WITH MODERATELY TO SEVERELY ACTIVE ULCERATIVE COLITIS IN THE LUCENT-1 INDUCTION TRIAL

**DOI:** 10.1093/jcag/gwac036.184

**Published:** 2023-03-07

**Authors:** S Danese, A Dignass, K Matsuoka, M Ferrante, M Long, I Redondo, T H Gibble, R Moses, N Morris, X Li, C Milch, M Abreu, J Jones

**Affiliations:** 1 Gastrointestinal immunopathology, Vita-Salute San Raffaele University - IRCCS San Raffaele Scientific Institute, Milan, Italy; 2 Agaplesion Markus Krankenhaus, Medizinische Klinik I, Frankfurt, Germany; 3 Gastroenterology and Hepatology, Tokyo Medical and Dental University, Tokyo, Japan; 4 Department of Gastroenterology and Hepatology, University Hospitals Leuven, Leuven, Belgium; 5 University of North Carolina at Chapel Hill, Chapel Hill, United States; 6 Produtos Farmacêuticos, Lda., Eli Lilly Portugal, Lisbon, Portugal; 7 Eli Lilly and Company, Indianapolis; 8 Miller School of Medicine, Crohn's and Colitis Center, University of Miami, Miami, United States; 9 Department of Medicine, Department of Community Health and Epidemiology, Dalhousie University, Halifax, Canada

## Abstract

**Background:**

Mirikizumab (miri), an anti-IL23/p19 monoclonal antibody, demonstrated efficacy compared with placebo (PBO) in the Phase 3, multicentre, randomized, double-blind LUCENT-1 induction study in patients with moderately to severely active ulcerative colitis (UC, NCT03518086).

**Purpose:**

This analysis assessed early onset of symptomatic improvement and symptomatic control during induction.

**Method:**

During the 12-week (W) induction study, 1162 adult patients (pts) with inadequate response, loss of response, or were intolerant to conventional therapy or biologic or tofacitinib therapy for UC, received miri IV Q4W (N=868) or PBO (N=294). We evaluated improvement for symptoms of stool frequency (SF), rectal bleeding (RB) and bowel movement urgency (BU), abdominal pain and fatigue. BU Numeric Rating Scale (NRS) change from baseline (BL), BU Clinical Meaningful Improvement (CMI), BU Remission, Fatigue NRS change from BL, Abdominal Pain Improvement, as well as SF Remission, RB Remission, Symptomatic Response and Symptomatic Remission were assessed.

**Result(s):**

As early as W2, miri-treated pts achieved a significantly greater reduction in RB subscores (p=0.001) and in SF subscores (p=0.035). From W2 and W4, a significantly greater percentage achieved SF Remission and RB Remission, respectively compared to PBO. A significantly greater percentage of miri-treated pts achieved Symptomatic Response compared to PBO from W2 (p=0.003) and of Symptomatic Remission compared with PBO from W4 (p<0.001). Miri-treated pts showed a significantly greater mean reduction in BU NRS scores as early as W2 compared to PBO (p=0.004). From W4, a significantly greater percentage of miri-treated pts achieved BU CMI versus PBO (p=0.044). From W7 onwards, a significantly greater percentage achieved BU Remission (p=0.002). The pts showed a significantly greater mean reduction in Fatigue NRS scores from W2 compared to PBO (p=0.014). As early as W4, a significant reduction of at least 30% in Abdominal Pain NRS score from BL was observed in the miri-treated pts compared with PBO (p=0.007). At W12, a significantly greater proportion of miri-treated pts achieved Symptomatic Response, Symptomatic Remission, RB Remission, SF Remission, BU change from BL, BU CMI and Remission, as well as Fatigue and Abdominal Pain Improvement, compared to PBO (Table).

**Image:**

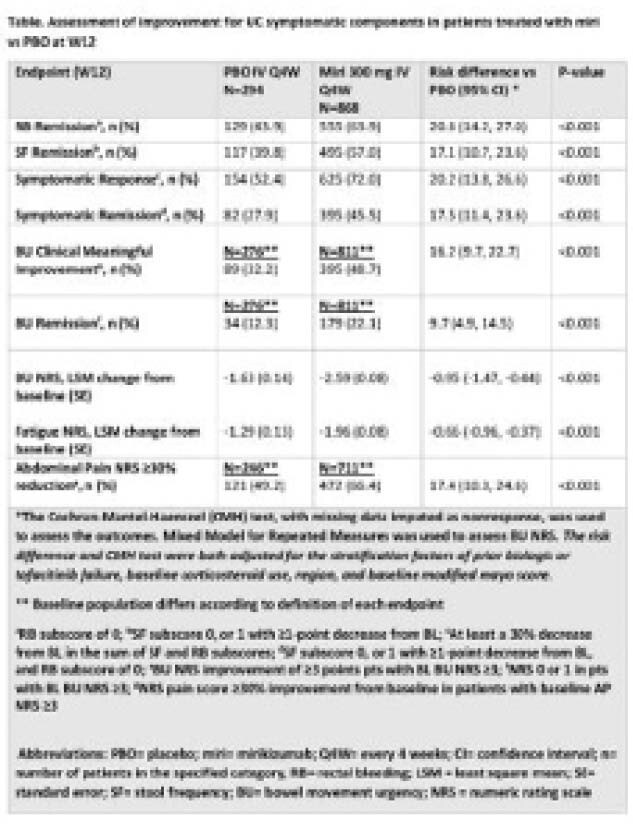

**Conclusion(s):**

Miri provides rapid control of UC symptoms, including BU and fatigue, as early as W2 compared with PBO in pts with moderately to severely active UC.

**Please acknowledge all funding agencies by checking the applicable boxes below:**

Other

**Please indicate your source of funding;:**

Eli Lilly and Company

**Disclosure of Interest:**

S. Danese Consultant of: AbbVie, Alimentiv, Allergan, Amgen, AstraZeneca, Athos Therapeutics, Biogen, Boehringer Ingelheim, Bristol Myers Squibb, Celgene, Celltrion, Dr. Falk Pharma, Eli Lilly and Company, Enthera, Ferring Pharmaceuticals, Gilead Sciences, Hospira, Inotrem, Janssen, Johnson & Johnson, Merck Sharp & Dohme, Mundipharma, Mylan, Pfizer, Roche, Sandoz Sublimity, Takeda, TiGenix, UCB Pharma, and Vifor Pharma, Speakers bureau of: AbbVie, Amgen, Ferring Pharmaceuticals, Gilead Sciences, Janssen, Mylan, Pfizer, and Takeda, A. Dignass Consultant of: AbbVie, Abivax, Amgen, Arena Pharmaceuticals, Bristol Myers Squibb (Celgene), Celltrion, Dr. Falk Pharma, Eli Lilly and Company, Ferring Pharmaceuticals, Fresenius Kabi, Galapagos, Gilead Sciences, Janssen, Merck Sharp & Dohme, Novartis, Pfizer, Pharmacosmos, Roche, Sandoz/Hexal, Takeda, Tillotts Pharma AG, and Vifor Pharma, Speakers bureau of: AbbVie, Amgen, Bristol Myers Squibb, Dr. Falk Pharma, Ferring Pharmaceuticals, Galapagos, High5Md, Janssen, Materia, Merck Sharp & Dohme, Pfizer, Sandoz, Takeda, Tillotts Pharma AG, and Vifor Pharma, K. Matsuoka Grant / Research support from: AbbVie, EA Pharma, JIMRO, Kissei Pharmaceutical, Kyowa Kyorin, Mitsubishi Tanabe, Mochida Pharmaceutical, and Zeria Pharmaceutical Nippon, Speakers bureau of: AbbVie, EA Pharma, JIMRO, Kissei Pharmaceutical, Kyowa Kyorin, Mitsubishi Tanabe, Mochida Pharmaceutical, Takeda, and Zeria Pharmaceutical Nippon, M. Ferrante Grant / Research support from: AbbVie, Amgen, Biogen, Janssen Cilag, Pfizer, Takeda, and Viatris, Consultant of: AbbVie, Boehringer Ingelheim, Celltrion, Eli Lilly and Company, Janssen Cilag, Medtronic, Merck Sharp & Dohme, Pfizer, Regeneron, Sandoz, Takeda, and Thermo Fisher Scientific, Speakers bureau of: AbbVie, Amgen, Biogen, Boehringer Ingelheim, Celltrion, Dr. Falk Pharma, Eli Lilly and Company, Ferring Pharmaceuticals, Janssen, Lamepro, Medtronic, Merck Sharp & Dohme, Mylan, Pfizer, Samsung Bioepis, Sandoz, Takeda, and Thermo Fisher Scientific, M. Long Consultant of: AbbVie, Bristol Myers Squibb, Calibr, Eli Lilly and Company, Genentech, Janssen, Pfizer, Prometheus Biosciences, Roche, Takeda, TARGET PharmaSolutions, and Theravance Biopharma, I. Redondo Employee of: Eli Lilly and Company, T. Gibble Employee of: Eli Lilly and Company, R. Moses Employee of: Eli Lilly and Company, N. Morris Employee of: Eli Lilly and Company, X. Li Employee of: Eli Lilly and Company, C. Milch Employee of: former employee, was employed at Eli Lilly and Company at the time of study, M. Abreu Grant / Research support from: Pfizer, Prometheus Biosciences, and Takeda, Consultant of: AbbVie, Arena Pharmaceuticals, Bristol Myers Squibb, Eli Lilly and Company, Gilead Sciences, Janssen, Microba Life Sciences, Prometheus Biosciences, UCB Pharma, and WebMD, Speakers bureau of: Alimentiv, Intellisphere LLC (HCP Live Institutional Perspectives in GI), Janssen, Prime CME, and Takeda, J. Jones: None Declared

